# PHILOS (Proximal Humerus Internal Locking System) Plating vs MultiLoc Nailing in Proximal Humerus Fracture: Systematic Review and Meta-Analysis of Radiological and Functional Outcomes

**DOI:** 10.7759/cureus.72251

**Published:** 2024-10-24

**Authors:** Jomon De Joseph, Aravind Gandhi P., Bijaya K Padhi

**Affiliations:** 1 Orthopedics, Mahatma Gandhi Medical College and Research Institute, Sri Balaji Vidyapeeth (Deemed-to-be-University), Puducherry, IND; 2 Community Medicine, School of Public Health, All India Institute of Medical Sciences, Nagpur, Nagpur, IND; 3 Community Medicine and School of Public Health, Postgraduate Institute of Medical Education and Research, Chandigarh, IND

**Keywords:** fragility fractures, nailing, orthopedics and trauma, philos plate, plating, proximal humeral fracture, shoulder injuries, s: osteoporosis

## Abstract

Proximal humeral fractures, predominantly affecting the elderly, pose significant treatment challenges due to the complex anatomy of the shoulder joint and variability in bone quality. MultiLoc nails (Synthes USA Products, West Chester, USA) are the latest construct, and PHILOS (Proximal Humerus Internal Locking System, (Synthes USA Products, West Chester, USA)) plates are the earlier construct used for the fixation of proximal humerus fractures. This systematic review aims to provide a comparison of MultiLoc nails and PHILOS plates, focusing on their effectiveness, safety, and patient outcomes. Our study included randomized controlled trials (RCTs) and observational studies comparing the effectiveness of MultiLoc nails and PHILOS plates. We searched literature in databases, including PubMed, EMBASE, and Web of Science, until December 20, 2023. The primary outcome of focus was the Neck Shaft Angle, supplemented by a range of secondary surgical and functional outcomes. The Nested Knowledge web software (Nested Knowledge, Inc., Saint Paul, USA) facilitated the screening and data extraction processes. The quality of included studies was assessed using the Cochrane Risk of Bias tool for RCTs (Cochrane, London, UK) and the ROBINS-I (Risk Of Bias In Non-randomised Studies - of Interventions) tool for observational studies (Cochrane, London, UK). R software version 4.3 (R Foundation for Statistical Computing, Vienna, Austria) was used to synthesize the collected data. Six studies met the inclusion criteria, primarily involving older adults in their mid-50s to late 70s. While MultiLoc nails offer shorter operation times and potentially reduced blood loss, both techniques effectively maintain the Neck Shaft Angle, a crucial factor for shoulder function. Comparable Constant-Murley scores and complication rates were observed between the two methods. PHILOS plates showed higher American Shoulder and Elbow Surgeons Shoulder Score (ASES) scores for two-part proximal humeral fractures with displacement. Other observations suggested advantages of MultiLoc nails in terms of faster union and fewer complications.

## Introduction and background

Proximal humeral fractures, commonly occurring skeletal injuries, predominantly affect the elderly and present significant treatment challenges [1-3]. These fractures occur in the upper portion of the arm bone near the shoulder and can greatly impair mobility and function. Their management complexity stems from the shoulder joint's intricate anatomy and the variability in bone quality, particularly in older patients [3]. Effective treatment of proximal humeral fractures is essential for restoring function and minimizing long-term complications that can severely affect patients' quality of life and independence [4].

Over time, the management strategies for proximal humeral fractures have evolved from conservative approaches like slings and physiotherapy to more advanced surgical interventions. Treatment choice depends on factors such as fracture type, patient age, activity level, and general health. Surgical options include plates and screws, intramedullary nails, and shoulder replacement for severe cases [5,6]. Each technique, with its specific indications and limitations, is continuously refined through clinical practice and research.

Among the surgical methods, using MultiLoc nails and plates has become increasingly prominent [7,8]. The MultiLoc nail system (Synthes USA Products, West Chester, USA), a more recent development, offers a minimally invasive approach aiming for stable fixation in complex fractures. It is known for preserving bone stock and reducing soft tissue damage. PHILOS plates (Synthes USA Products, West Chester, USA), however, have been a fundamental aspect of orthopedic surgery for a longer duration. They provide robust fixation and are often favored in specific proximal humeral fracture scenarios [9]. While each method carries unique potential complications and varying success rates, as recent studies and clinical practice indicate, their development reflects the dynamic nature of orthopedic surgery and the ongoing effort to enhance patient outcomes [9].

Despite advancements in surgical techniques, there is a notable gap in the literature concerning an in-depth comparison between the MultiLoc nail system and PHILOS plates for treating proximal humeral fractures. This systematic review and meta-analysis aim to bridge this gap by thoroughly analyzing existing studies comparing these two methods. The goal is to compile evidence to inform clinical decision-making and optimize patient care. This review intends to evaluate each technique's efficacy, safety, and patient outcomes, offering evidence-based recommendations for orthopedic surgeons. Ultimately, this systematic review and meta-analysis seek to deepen the understanding of these surgical options, enhancing treatment strategies and improving patient quality of life in managing proximal humeral fractures.

## Review

Methods

Study Design and Registration

The study represents a collaborative effort between three institutions. This collaboration integrated expertise across institutions, strengthening the research. This systematic review and meta-analysis were designed to compare the outcomes of MultiLoc nail and PHILOS plate treatments in proximal humeral fractures. PRISMA (Preferred Reporting Items for Systematic Reviews and Meta-Analysis) guidelines were followed for this study given in Table 1. Our objective was to analyze data from various studies to evaluate efficacy, safety, and patient outcomes. The protocol for this review is registered with PROSPERO (CRD42024523119).

**Table 1:** PRISMA Checklist Source: Page et al. [10] PRISMA: Preferred Reporting Items for Systematic Reviews and Meta-Analysis

Section and Topic	Item #	Checklist item	Location where item is reported
TITLE			
Title	1	Identify the report as a systematic review.	1
ABSTRACT			
Abstract	2	See the PRISMA 2020 for Abstracts checklist. (made as per the Journal guidelines)	2
INTRODUCTION			
Rationale	3	Describe the rationale for the review in the context of existing knowledge.	3
Objectives	4	Provide an explicit statement of the objective(s) or question(s) the review addresses.	3
METHODS			
Eligibility criteria	5	Specify the inclusion and exclusion criteria for the review and how studies were grouped for the syntheses.	3
Information sources	6	Specify all databases, registers, websites, organisations, reference lists and other sources searched or consulted to identify studies. Specify the date when each source was last searched or consulted.	3
Search strategy	7	Present the full search strategies for all databases, registers and websites, including any filters and limits used.	Table 3
Selection process	8	Specify the methods used to decide whether a study met the inclusion criteria of the review, including how many reviewers screened each record and each report retrieved, whether they worked independently, and if applicable, details of automation tools used in the process.	4
Data collection process	9	Specify the methods used to collect data from reports, including how many reviewers collected data from each report, whether they worked independently, any processes for obtaining or confirming data from study investigators, and if applicable, details of automation tools used in the process.	4
Data items	10a	List and define all outcomes for which data were sought. Specify whether all results that were compatible with each outcome domain in each study were sought (e.g., for all measures, time points, analyses), and if not, the methods used to decide which results to collect.	4, Table 1
	10b	List and define all other variables for which data were sought (e.g., participant and intervention characteristics, funding sources). Describe any assumptions made about any missing or unclear information.	4
Study risk of bias assessment	11	Specify the methods used to assess risk of bias in the included studies, including details of the tool(s) used, how many reviewers assessed each study and whether they worked independently, and if applicable, details of automation tools used in the process.	4,
Effect measures	12	Specify for each outcome the effect measure(s) (e.g. risk ratio, mean difference) used in the synthesis or presentation of results.	5
Synthesis methods	13a	Describe the processes used to decide which studies were eligible for each synthesis (e.g. tabulating the study intervention characteristics and comparing against the planned groups for each synthesis (item #5)).	5, Table 1
	13b	Describe any methods required to prepare the data for presentation or synthesis, such as handling of missing summary statistics, or data conversions.	NA
	13c	Describe any methods used to tabulate or visually display results of individual studies and syntheses.	5
	13d	Describe any methods used to synthesize results and provide a rationale for the choice(s). If meta-analysis was performed, describe the model(s), method(s) to identify the presence and extent of statistical heterogeneity, and software package(s) used.	5
	13e	Describe any methods used to explore possible causes of heterogeneity among study results (e.g. subgroup analysis, meta-regression).	5
	13f	Describe any sensitivity analyses conducted to assess robustness of the synthesized results.	5
Reporting bias assessment	14	Describe any methods used to assess risk of bias due to missing results in a synthesis (arising from reporting biases).	NA
Certainty assessment	15	Describe any methods used to assess certainty (or confidence) in the body of evidence for an outcome.	NA
RESULTS			
Study selection	16a	Describe the results of the search and selection process, from the number of records identified in the search to the number of studies included in the review, ideally using a flow diagram.	5, Figure-1, 2
	16b	Cite studies that might appear to meet the inclusion criteria, but which were excluded, and explain why they were excluded.	5, Table 1
Study characteristics	17	Cite each included study and present its characteristics.	Table 1
Risk of bias in studies	18	Present assessments of risk of bias for each included study.	Table 4
Results of individual studies	19	For all outcomes, present, for each study: (a) summary statistics for each group (where appropriate) and (b) an effect estimate and its precision (e.g. confidence/credible interval), ideally using structured tables or plots.	Table 1, Figure 2, 3
Results of syntheses	20a	For each synthesis, briefly summarise the characteristics and risk of bias among contributing studies.	5
	20b	Present results of all statistical syntheses conducted. If meta-analysis was done, present for each the summary estimate and its precision (e.g. confidence/credible interval) and measures of statistical heterogeneity. If comparing groups, describe the direction of the effect.	5, 6 Figure 2, 3
	20c	Present results of all investigations of possible causes of heterogeneity among study results.	5, 6
	20d	Present results of all sensitivity analyses conducted to assess the robustness of the synthesized results.	6
Reporting biases	21	Present assessments of risk of bias due to missing results (arising from reporting biases) for each synthesis assessed.	NA
Certainty of evidence	22	Present assessments of certainty (or confidence) in the body of evidence for each outcome assessed.	NA
DISCUSSION			
Discussion	23a	Provide a general interpretation of the results in the context of other evidence.	6
	23b	Discuss any limitations of the evidence included in the review.	7
	23c	Discuss any limitations of the review processes used.	7
	23d	Discuss implications of the results for practice, policy, and future research.	7
OTHER INFORMATION			
Registration and protocol	24a	Provide registration information for the review, including register name and registration number, or state that the review was not registered.	3
	24b	Indicate where the review protocol can be accessed, or state that a protocol was not prepared.	3
	24c	Describe and explain any amendments to information provided at registration or in the protocol.	NA
Support	25	Describe sources of financial or non-financial support for the review, and the role of the funders or sponsors in the review.	8
Competing interests	26	Declare any competing interests of review authors.	8
Availability of data, code and other materials	27	Report which of the following are publicly available and where they can be found: template data collection forms; data extracted from included studies; data used for all analyses; analytic code; any other materials used in the review.	8

Inclusion and Exclusion Criteria

We included studies such as randomized controlled trials (RCTs), cohort studies, and case-control studies that directly compared MultiLoc nails with PHILOS plates in treating proximal humeral fractures. We specifically focused on studies involving only these two methods; interventions using any nail system other than MultiLoc were excluded. The target population for our review was individuals with proximal humeral fractures. The primary outcome we were interested in was NSA (Neck Shaft Angle). Secondary outcomes of interest included Volumes of Blood Loss, American Shoulder and Elbow Surgeons Score (ASES), Union Time, Constant-Murley Score, Visual Analog Scale (VAS), and Duration of Surgery. Studies reporting on any of these outcomes were considered an inclusion. Exclusions applied to studies not making a direct comparison between MultiLoc nails and PHILOS plate, as well as animal studies, case reports, editorials, opinion pieces, and articles not published in English. We imposed no restrictions regarding the type of hospital or geographical region of the study.

Search Strategy

A literature search was performed to identify relevant articles for this systematic review. Our search strategy was comprehensive, encompassing several major databases: PubMed, EMBASE, and the Web of Science. The timeframe for the search extended from the inception of these databases to November 20, 2023, which was later updated on December 20, 2023. Keywords used in the search included various combinations and synonyms of "proximal humeral fractures," "MultiLoc nails," and "locking plates." This strategy was crafted to be broad enough to include all pertinent studies while maintaining a specific focus on comparing the two surgical interventions. No filters were applied during the search, ensuring a thorough capture of relevant literature. The detailed search strategy is provided in Table 2.

**Table 2:** The adjusted search terms as per searched electronic databases

Database	Search Query	Results
PubMed	"proximal humeral fractures"[MeSH Terms] OR "proximal humeral fractures"[All Fields] OR "shoulder fractures"[All Fields] OR "humeral head fractures"[All Fields]) AND ("Multiloc nails"[All Fields] OR "intramedullary nailing"[MeSH Terms] OR "intramedullary nailing"[All Fields] OR "intramedullary nail"[All Fields] OR philos OR "locking plates"[All Fields] OR "orthopedic fixation devices"[All Fields] OR "philos plate"[All Fields])	548
Embase	('proximal humeral fractures' OR 'shoulder fractures' OR 'humeral head fractures') AND ('multiloc nails' OR 'intramedullary nailing' OR 'intramedullary nail' OR philos OR 'locking plates' OR 'orthopedic fixation devices' OR 'philos plate')	525
Web of Science	("proximal humeral fractures" OR "shoulder fractures" OR "humeral head fractures") AND ("Multiloc nails" OR "intramedullary nailing" OR "intramedullary nail" OR philos OR "locking plates" OR "orthopedic fixation devices" OR "philos plate") (All Fields)	310

Screening of Articles

After conducting the literature search, we processed the collected records to remove duplicates using a semi-automated software named Nested Knowledge (Nested Knowledge, Inc., Saint Paul, USA). The initial screening of these records was independently performed by two reviewers. This screening process was divided into two distinct phases: first, we scrutinized titles and abstracts, followed by a thorough examination of the full texts. Two independent reviewers undertook the screening tasks. In cases where there were differences in the reviewers' assessments, a consensus-building approach was employed. To resolve any discrepancies, a third reviewer with expertise in the subject matter was consulted.

Data Extraction

The data extraction process was conducted by two independent reviewers using a pre-defined and standardized form, which was carefully crafted to capture essential information from each study systematically. The extracted data included a comprehensive range of elements such as the study design, sample size, participant demographics, detailed descriptions of the fractures and treatment methods, the duration of follow-up, and the specific outcomes measured. Additionally, we recorded the authors' names, the country where the study was conducted, the average age of participants, and the overall sample size. For every outcome measured, the means and standard deviations were meticulously extracted to provide a thorough quantitative analysis. To facilitate the data extraction process, we utilized the tagging feature of the Nested Knowledge software, which allowed for efficient and organized data management. Subsequently, the extracted data was converted to Microsoft Excel (Microsoft Corporation, Redmond, USA) for further analysis and synthesis.

Quality Assessment

The quality of each included study was critically appraised (Tables 3, 4).

**Table 3:** Quality assessment of non-randomized studies by ROBINS-I Domains: D1- Bias due to confounding, D2-Bias due to selection of participants, D3-Bias in classification of interventions, D4-Bias due to deviations from intended intervention, D5-Bias due to missing data, D7-Bias in selection of the reported result ROBINS-I: Risk Of Bias In Non-randomised Studies - of Interventions by Cochrane, London, UK.

Study	D1	D2	D3	D4	D5	D6	D7	Overall
Bu et al. [11]	Moderate	Moderate	Low	Low	Moderate	Moderate	Moderate	Moderate
Gomes et al. [12]	Moderate	Moderate	Moderate	Low	Moderate	Moderate	Moderate	Serious
Li et al. [13]	Low	Moderate	Low	No info	Moderate	Moderate	No info	Serious
Zhu et al. [14]	No information	No information	Low	Low	Low	Low	Low	No information

**Table 4:** Quality assessment of RCTs by Cochrane RoB-2 Domains: D1: Bias arising from the randomization process. D2: Bias due to deviations from intended intervention. D3: Bias due to missing outcome data. D4: Bias in measurement of the outcome. D5: Bias in selection of the reported result RoB-2: Version 2 of the Cochrane risk-of-bias tool for randomized trials by Cochrane, London, UK.

Study	D1	D2	D3	D4	D5	Overall
Helfen et al. [9]	Low	Some concern	Some concern	Low	Some concern	Some concerns
Wu et al. [15]	Some concerns	Low	Low	Low	Low	Some concerns

Statistical Analysis

Data were synthesized using a random-effects model in the meta-analysis to account for between-study variability. For continuous outcome variables, means and standard deviations for the MultiLoc and PHILOS groups were pooled according to their respective sample sizes. Heterogeneity among studies was assessed using the I2 statistic. I² value of 0% indicated the absence of observed heterogeneity, while higher values indicated increasing levels of heterogeneity, with 25% considered as low, 50% as moderate, and 75% as high [16]. The tau-squared value was computed using maximum likelihood estimation to gain further insights into the heterogeneity [16,17]. To evaluate publication bias, funnel plots were employed, and they were quantitatively assessed using Egger's regression test. Typically, a p-value below 0.05 was considered statistically significant. All statistical analyses were conducted using R software Version 4.3 (R Foundation for Statistical Computing, Vienna, Austria).

Results

Literature Search

**Figure 1:**
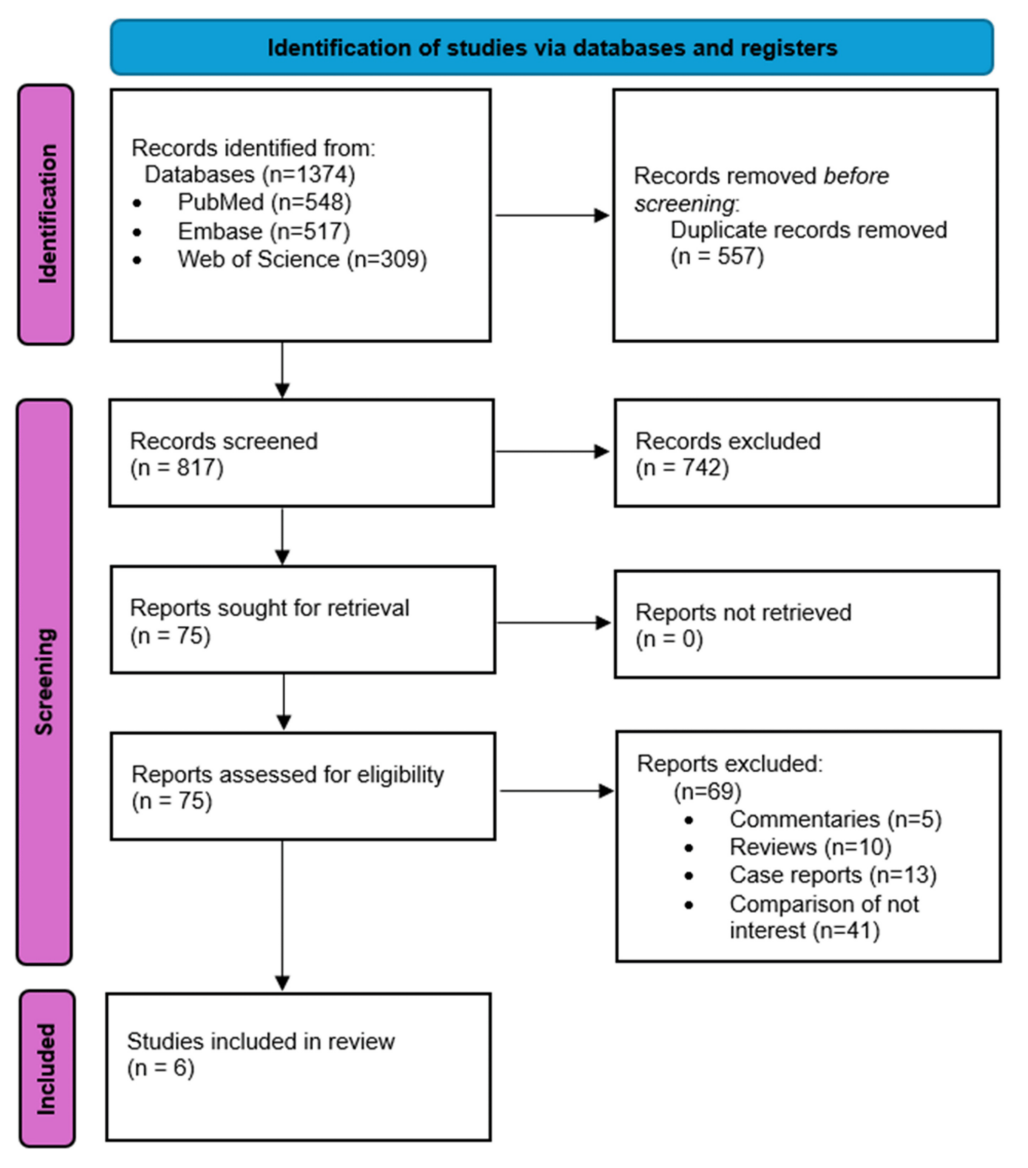
PRISMA flowchart showing the study selection process and studies included in the systematic review PRISMA: Preferred Reporting Items for Systematic Reviews and Meta-Analysis

The selection process for studies in a systematic review, as presented in the provided PRISMA flow diagram (Figure 1), began with the identification of 1,621 records from various databases: 548 from PubMed, 517 from Embase, and 309 from Web of Science. Initially, 557 duplicate records were removed. Then, 817 records underwent screening, after which 742 were excluded, leaving 75 records that were suitable for retrieval and further assessment. No reports were left unretrieved. Upon closer examination, 69 full-text reports were excluded for specific reasons: five were commentaries, 10 were review articles, 13 were case reports, and 41 did not feature the comparisons of interest. The remaining six studies passed the eligibility criteria and were included in the final qualitative analysis and meta-analysis.

Characteristics of Included Studies

The studies encompass a range of designs, including retrospective analyses and RCTs, conducted across countries like China, Brazil, and Germany. Participants were predominantly older adults with average ages ranging from the mid-50s to late 70s. Sample sizes varied from 17 to 115. Common outcomes measured across these studies were the neck shaft angle (NSA), volumes of blood loss, and functional scores like ASES and Constant-Murley scores. The studies were of low to moderate quality (Tables 3, 4).

The studies included in the analysis presented distinct findings regarding the comparison between MultiLoc nails and PHILOS plates for the treatment of proximal humeral fractures. Bu et al. (2021) found that MultiLoc has several advantages over the PHILOS plate, including reduced blood loss, shorter operation time, faster union, and fewer complications. It also preserved the Neck Shaft Angle (NSA) better at the final follow-up [11]. Zhu et al. (2021) highlighted that the MultiLoc nail led to better recovery of the humeral NSA, lower Visual Analogue Scale (VAS) pain scores one month after surgery, and a significantly lower incidence of adverse reactions compared to the PHILOS plate [14]. Wu et al. (2021) showed that the MultiLoc nail was associated with shorter surgical durations, less intraoperative hemorrhage, and better functional outcomes at 6 months post-surgery [15]. On the other hand, Helfen et al. (2020) demonstrated that the PHILOS plate was associated with better Disabilities of the Arm, Shoulder and Hand (DASH) scores, higher Constant-Murley, and higher Oxford Shoulder Scores than MultiLoc [9]. However, the ASES scores and the rates of revision surgery were similar between the two groups. Li et al. (2020) reported identical superior shoulder function rates between the two groups at 12 months, despite the MultiLoc group showing some advantages in terms of operation time and lower intraoperative bleeding volume [13]. Gomes et al. (2022) showed that there was no significant difference in complication rates between nailing and plating, though the nail group experienced less varus loss [12]. These findings suggest that while both MultiLoc nails and PHILOS plates are effective for treating proximal humeral fractures, there may be specific advantages to each method depending on the outcome of interest. For instance, the MultiLoc nail might be preferable for reducing operation time and blood loss, whereas the PHILOS plate could be more beneficial for certain functional outcomes.

Meta-Analysis of NSA

**Figure 2:**
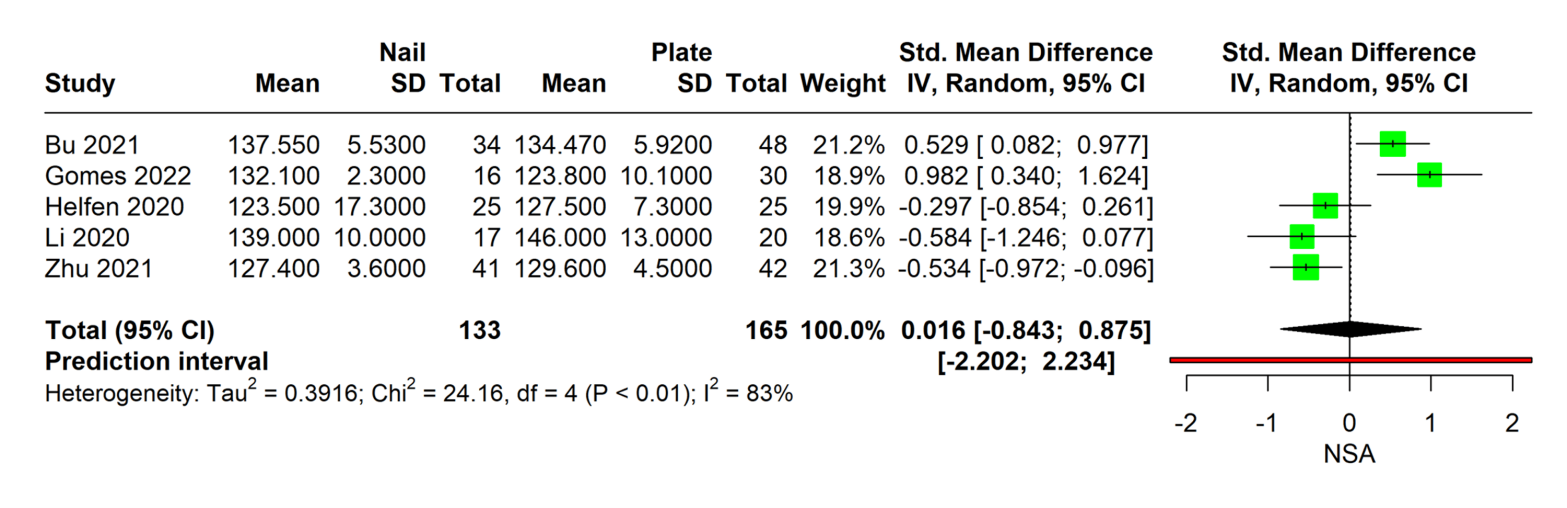
Forest plot comparing NSA Between MultiLoc nail and PHILOS plate surgeries Studies with NSA: Bu 2021 [11], Gomes 2022 [12], Helfen 2020 [9], Li 2020 [13], Zhu 2021 [14] NSA: Neck Shaft Angle

In this meta-analysis, we compared the efficacy of MultiLoc nails versus PHILOS plates in maintaining the neck shaft angle (NSA) after proximal humerus fracture surgery. The analysis included a total of 133 patients who underwent surgery with MultiLoc nail, and 165 patients treated with PHILOS plate across five studies. The pooled results revealed a high level of heterogeneity (I² = 83%), indicating considerable variability in NSA outcomes between studies. The mean difference in NSA was not statistically significant, with a standardized mean difference (SMD) of 0.016 (95%CI: -0.843 to 0.875). The comprehensive p-value of 0.96 further indicates no significant difference in the ability to preserve the NSA between the two surgical interventions (Figure 2).

Meta-Analysis of Duration of Surgery

**Figure 3:**
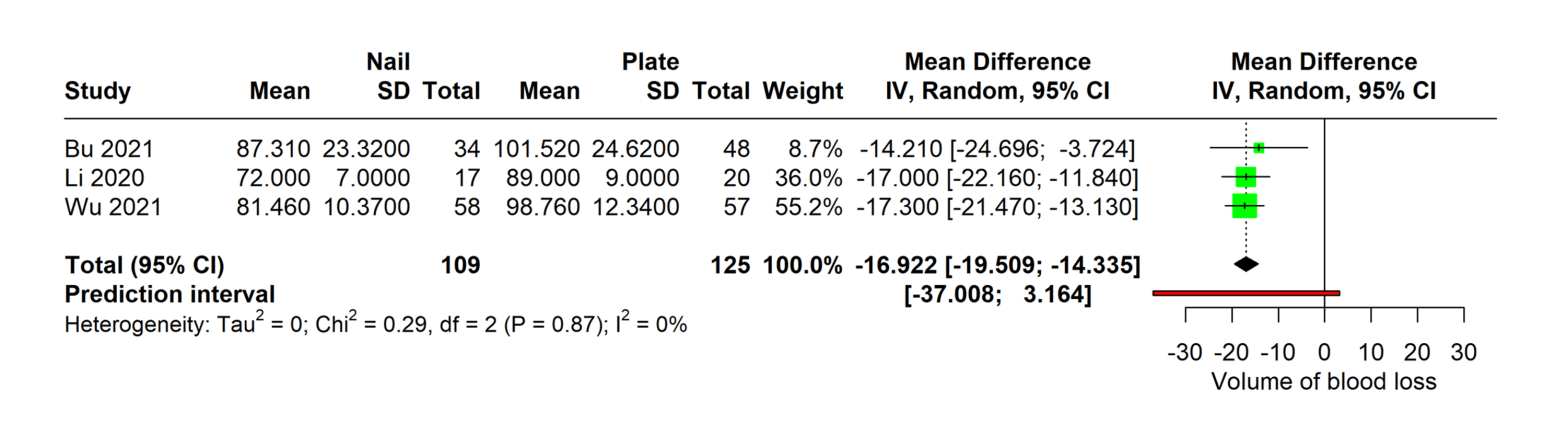
Forest plot comparing duration of surgery with MultiLoc nail versus PHILOS plate Studies with duration of surgery: Bu 2021 [11], Li 2020 [13], Wu 2021 [15]

In a pooled analysis of three studies comparing surgical durations for procedures utilizing MultiLoc versus PHILOS plates, the data revealed a consistent reduction in operation time with the use of MultiLoc nails. Three studies encompassed a total of 109 patients in the nail group and 125 patients in the plate group, showing no heterogeneity in surgical duration outcomes (I² = 0%). The mean surgery duration for the nail group was significantly less by an average of 16.922 minutes (95% CI: -19.50 to -14.335, p=0.001) compared to the PHILOS group (Figure 3).

Meta-Analysis of Volume of Blood Loss

**Figure 4:**
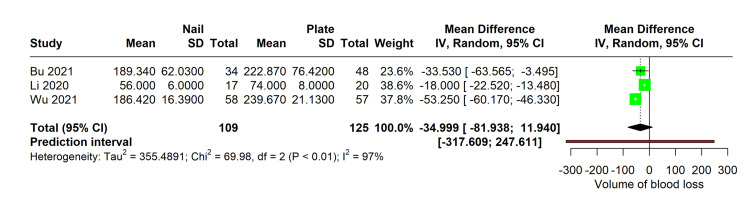
Forest plot comparing volume of blood loss between MultiLoc nail and PHILOS plate surgeries Studies with volume of blood loss: Bu 2021 [11], Li 2020 [13], Wu 2021 [15]

In a meta-analysis of three studies comparing blood loss during surgical procedures employing MultiLoc nails versus PHILOS plates, the evidence demonstrated no significant difference in the volume of blood loss between the two methods. The meta-analysis included a total of 109 patients in the MultiLoc nail group and 125 in the PHILOS plate group. Despite the marked heterogeneity observed among the studies (I² = 97%), the pooled mean difference in blood loss was -34.999 mL (95% CI: -81.938 to 11.940), and a p-value of 0.08, indicating a non-significant trend towards reduced blood loss in the MultiLoc nail group (Figure 4).

Publication Bias

The assessment of publication bias, which refers to the tendency for studies with positive results to be more likely to be published, was limited in our analysis. Given the small number of studies included, it was not feasible to conduct robust statistical tests, such as funnel plot asymmetry or Egger's regression, to detect publication bias reliably. This limitation is significant because publication bias can lead to an overestimation or underestimation of the true effect size. The inability to evaluate publication bias suggests caution when interpreting the results of our meta-analysis.

Discussion

Our systematic review and meta-analysis provide a comprehensive comparison between MultiLoc nails and PHILOS plates for the treatment of proximal humeral fractures. The findings shed light on the relative advantages and disadvantages of these surgical interventions, offering insights that could guide clinical decision-making. The NSA maintenance, a critical outcome for ensuring proper shoulder function, showed no significant difference between MultiLoc nails and PHILOS plates. This finding suggests that both techniques are equally effective in preserving the anatomical structure of the proximal humerus post-surgery. However, the high level of heterogeneity in these results indicates that individual patient factors, such as bone quality and fracture type, may significantly influence the outcome. Therefore, surgeons should consider these factors when choosing the appropriate surgical method. In terms of operation time, our analysis indicated a consistent reduction in duration with the use of MultiLoc nails. This efficiency could be attributed to the less invasive nature of the nail system. Shorter operation times not only benefit the surgeon but may also reduce the patient's exposure to anesthesia and potentially lower the risk of perioperative complications. Regarding blood loss, our findings suggested a non-significant trend towards reduced blood loss in the MultiLoc nail group. While not statistically significant, this trend may have clinical relevance, especially in elderly patients or those with comorbidities where minimizing blood loss is crucial. This aspect, coupled with shorter operation times, positions the MultiLoc nail as a potentially more favorable option in specific patient populations. A study by Dominik Malcherczyk et al. says fracture classification does not affect blood loss but higher transfusion rates are associated with longer duration of surgery [18]. 

Although our comparison specifically focused on the MultiLoc nail versus the PHILOS plate, which are relatively newer generations, intramedullary nails, and locking plates have been studied previously. For example, a prior systematic review by Wang et al. [19] examined locking plates and intramedullary nails for proximal humeral fractures. They concluded that both methods effectively manage displaced two-, three-, and four-part proximal humeral fractures in elderly patients. They observed comparable Constant-Murley scores and complication rates between the two methods. A notable advantage of the locking plate was its higher ASES score for two-part proximal humeral fractures with displacement. In another meta-analysis, Li et al. [20] demonstrated that intramedullary nails were superior to locking plates in aspects such as shorter incision lengths, reduced peri-operative bleeding duration, faster operation times, and quicker fracture healing periods. However, both treatments had similar results regarding Constant-Murley scores and the frequency of postoperative complications.

Our analysis offers vital clinical implications. Firstly, the equivalent effectiveness of both methods in maintaining the NSA allows clinicians to base their surgical choice on other considerations, such as patient preference and resource availability, without compromising the anatomical outcome. NSA has been observed to be not influenced by the type of fracture but is based on the fracture reduction [21]. The observed high heterogeneity in NSA outcomes highlights the need for personalized treatment plans, considering patient-specific factors like bone quality and fracture type. The operational efficiency of MultiLoc nails demonstrated through shorter operation times, is particularly beneficial in reducing perioperative risks, especially in older or medically complex patients. While not statistically significant, the trend towards reduced blood loss with MultiLoc nails warrants consideration in scenarios where blood conservation is critical. Additionally, insights from previous studies on intramedullary nails and locking plates provide a broader context for treatment choices, such as the preference for locking plates in certain fracture types due to higher ASES scores. These findings not only guide clinical decision-making but also point to areas for future research and implications for surgical training and resource allocation. Ultimately, the choice between MultiLoc nails and PHILOS plates should be a balanced decision based on a comprehensive evaluation of individual patient needs, surgical efficiency, and the potential for reduced complications, thereby enhancing patient outcomes and optimizing healthcare resources in orthopedic surgery.

Future studies should emphasize long-term outcomes, focusing on the durability and longevity of these interventions. The need for prospective RCTs is paramount in providing more definitive evidence about the relative merits of each treatment option. A patient-centered approach, including assessments of pain, quality of life, and functional recovery, is crucial for tailoring treatments to individual needs. Detailed subgroup analyses based on diverse patient characteristics will offer deeper insight into which groups benefit most from each surgical intervention. Additionally, evaluating the cost-effectiveness of these treatments is essential, particularly in resource-constrained settings. Research should also delve into refining surgical techniques and developing comprehensive training programs for surgeons, adapting to new technologies and methods. A thorough comparative analysis of the complication profiles of MultiLoc nails and PHILOS plates will aid in understanding and preventing specific complications. Biomechanical studies can provide valuable insights into the implants' mechanical properties, influencing surgical decisions. A global perspective on treatment efficacy, considering variations in healthcare systems and demographics, is also vital. Integrating emerging technologies such as 3D printing and AI-based predictive models can revolutionize the treatment landscape of proximal humeral fractures [22]. These future research directions will build upon our findings, contribute to the orthopedic surgery body of knowledge, and enhance patient care strategies.

Limitations

Our study possesses several strengths along with a few limitations. Firstly, we strictly adhered to the PRISMA guidelines, ensuring a high standard of research methodology and reporting. Additionally, every stage of the review process involved at least two independent reviewers, enhancing the objectivity and reliability of our evaluations. Our team was composed of a multidisciplinary group, including subject matter experts, which brought a diverse and comprehensive perspective to the analysis. A significant advantage of our approach was the ability to perform a meta-analysis, allowing for a quantitative synthesis of results, and providing clearer insights into the effectiveness of the treatments studied. However, the study was not without limitations. One major constraint was the limited number of studies available for inclusion. This scarcity of data could potentially impact the breadth and generalizability of our conclusions. Moreover, a considerable portion of the studies included were retrospective. While these studies offer valuable insights, they generally have a lower level of evidence compared to prospective studies. Retrospective studies often come with inherent biases and limitations in data availability, which could influence the outcomes of our analysis. The present review is also limited by the lack of detailed information on factors known to influence the outcome of surgical fixation for proximal humerus neck fractures. These factors include the type of fracture, patient comorbidities and BMI, steroid use, bone quality, and smoking status. This indicates a major lacuna, which needs to be worked out in future studies. Despite these limitations, our study analyzed factors like shorter operation times and neck shaft angle which contribute valuable knowledge to the field and aid in informed decision-making for the treatment of proximal humeral fractures.

## Conclusions

Both MultiLoc nails and PHILOS plates are effective in treating proximal humeral fractures. While MultiLoc nails offer shorter operation times and potentially reduced blood loss, both techniques effectively maintain the Neck Shaft Angle, a crucial factor for shoulder function. The choice between these methods should be tailored to individual patient needs, considering factors such as bone quality, fracture type, and available resources. Future research should focus on long-term outcomes, functional recovery, and patient-centered measures to provide definitive evidence and inform healthcare decision-making.
